# Enhanced Correlation between Arousal and Infra-Slow Brain Activity in Experienced Meditators

**DOI:** 10.3390/brainsci14100981

**Published:** 2024-09-27

**Authors:** Duho Sihn, Sung-Phil Kim

**Affiliations:** Department of Biomedical Engineering, Ulsan National Institute of Science and Technology, Ulsan 44919, Republic of Korea

**Keywords:** arousal, EEG, GSR, infraslow activity, meditation, phase synchronization

## Abstract

Background/Objectives: Meditation induces changes in the nervous system, which presumably underpin positive psychological and physiological effects. Such neural changes include alterations in the arousal fluctuation, as well as in infraslow brain activity (ISA, <0.1 Hz). Furthermore, it is known that fluctuations of arousal over time correlate with the oscillatory phase of ISA. However, whether this arousal–ISA correlation changes after meditation practices remains unanswered.; Methods: The present study aims to address this question by analyzing a publicly available electroencephalogram (EEG) dataset recorded during meditation sessions in the groups of experienced meditators and novices. The arousal fluctuation is measured by galvanic skin responses (GSR), and arousal–ISA correlations are measured by phase synchronization between GSR and EEG ISAs.; Results: While both groups exhibit arousal–ISA correlations, experienced meditators display higher correlations than novices. These increased arousal–ISA correlations in experienced meditators manifest more clearly when oscillatory phase differences between GSR and EEG ISAs are either 0 or π radians. As such, we further investigate the characteristics of these phase differences with respect to spatial distribution over the brain. We found that brain regions with the phase difference of either 0 or π radians form distinct spatial clusters, and that these clusters are spatially correlated with functional organization estimated by the principal gradient, based on functional connectivity.; Conclusions: Since increased arousal–ISA correlations reflect enhanced global organization of the central and autonomic nervous systems, our findings imply that the positive effects of meditation might be mediated by enhanced global organization of the nervous system.

## 1. Introduction

Meditation has beneficial psychological and physiological effects. Regular practice of meditation improves cognitive abilities [1], reduces stress [2,3,4], and relieves symptoms of psychiatric disorders [5,6]. These effects generally manifest after prolonged practice periods. All of these consequences of meditation indicate that meditation can alter the central and autonomic nervous systems (CNS and ANS). Prior neuroscience research has supported this by showing that interactions between CNS and ANS are increased after meditation practices [7]. Moreover, studies have revealed that meditation lowers the arousal level [8,9]. Yet, neural mechanisms mediating the practice of meditation and changes in the arousal level remain largely unknown.

Fluctuations of the arousal levels over time correlate with slowly changing brain activity. One such brain activity is infraslow activity (ISA), which refers to very slow temporal changes of the brain oscillations in a frequency band between 0.01 and 0.1 Hz [10,11]. Fluctuations of the arousal levels correlate with the amplitude of the ISA of blood-oxygen-level-dependent (BOLD) signals measured by functional magnetic resonance imaging (fMRI) [12,13], and the oscillatory phase of the ISA of electroencephalograms (EEG) [14]. Furthermore, both EEG ISA and BOLD ISA are correlated with each other [15,16]. Since meditation alters the arousal level, it may also affect correlations between ISA and the arousal fluctuation, which still remains unexplored.

Studies have demonstrated that the EEG ISA of experienced meditators differs from that of novices. Experienced meditators exhibit lower levels of EEG ISA phase synchronization [17] and amplitudes [18] compared to novices. Furthermore, previous research suggests that increases in correlations between the arousal fluctuation and the oscillatory phase of BOLD ISA represent enhanced global organization of CNS and ANS [12]. Given that such enhanced global organization has been associated with the remission of psychiatric disorders [19,20,21], increased correlations between the arousal fluctuation and the oscillatory phase of ISA could be crucial for the therapeutic effectiveness of meditation. Correlations between CNS and ANS are increased after mediation practices in the fast frequency ranges (>1 Hz). For example, EEG theta power is positively correlated with the high-frequency heart rate variability (HRV), which indicates the arousal level [7]. Additionally, the amplitude of heartbeat-evoked potentials in experienced meditators is smaller than in novices [22]. Yet, as the arousal level fluctuates in the infraslow frequency [14], it would be important to find correlations between CNS and ANS via neurophysiological signals that fluctuate at the infraslow frequency. As for CNS, ISA can represent this infraslow frequency fluctuation of brain activity. As for ANS, the arousal level fluctuations can be measured by many modalities, including HRV [7] and galvanic skin response (GSR) [23,24]. In particular, GSR has been most widely used to monitor the changes of the arousal level over time [25,26]. In addition, it is noteworthy that the significance of correlations between various physiological measures of arousal is task-dependent [27], which indicates that investigations using physiological measures other than cardiac activity are needed to support the generality of a global organizational hypothesis for meditation. Therefore, we aimed to study correlations between CNS and ANS in the infraslow frequency band using ISA and GSR.

The present study aims to investigate whether correlations between the arousal fluctuation and EEG ISA (arousal–ISA correlation) increase after meditation practices. To this end, we analyzed a publicly available dataset [28] in which the groups of experienced meditators and novices practiced Himalayan yoga meditation. In this dataset, the arousal fluctuation was probed using GSR, a well-known indicator of physiological arousal [23,24]. Specifically, the arousal fluctuation was measured using tonic GSR, since the amplitude of tonic GSR is known to represent the arousal level [25,26]. Yet, we focused on the arousal fluctuation, not the arousal level, per se. This makes the phase of GSR oscillations more important than the amplitude of them for the analysis of the arousal fluctuation. Therefore, in line with our previous study, we used phase synchronization between EEG and GSR to investigate arousal–ISA correlations [14]. Furthermore, we have reported that the meditation effect on EEG ISA varied with a range of infraslow frequency bands [18]. Hence, for arousal–ISA correlations, we calculated phase synchronization between the oscillatory phase of EEG ISA and GSR for various infraslow frequencies ranging from 0.01 to 0.1 Hz. Here, weaker phase synchronization indicated a lower arousal–ISA correlation. We then tested whether the degree of phase synchronization was different between the experienced meditator and Novice groups.

## 2. Materials and Methods

### 2.1. Dataset

We analyzed an open dataset, publicly available at: https://openneuro.org/datasets/ds001787/versions/1.0.3 (accessed on 22 May 2021) [28]. This dataset includes EEG and GSR data from 12 experienced meditators (Expert; 3 females) and 12 novices (Novice; 10 females) practicing Himalayan yoga meditation. The mean ages were 39.3 ± 12.0 years old for the Expert group and 45.0 ± 14.8 years old for the novices. We confirmed no significant difference in age between the groups (two-tailed Wilcoxon rank-sum test, *p* > 0.05). The experiments lasted between 45 and 90 min. The Expert group included participants that performed the meditation practice for more than 2 h every day and sustained this practice for more than 1 year. Otherwise, participants were included in the Novice group. Participants practiced meditation according to the following instructions: They began the meditation with a mental body scan in a motionless seated position, followed by the mental recitation of a mantra. The recitation of a mantra is considered to make the mind stable without the intentional focusing of attention [29].

Participants were engaged in repeated meditation sessions while 64-channel EEG and single-channel GSR signals were simultaneously recorded using a DC-coupled amplifier (ActiveTwo, BioSemi, Amsterdam, The Netherlands) at a sampling rate of 256 Hz [29]. The raw data was recorded under the “CMS/DRL” system of BioSemi, in which the locations of ground and reference electrodes were not critical (https://www.biosemi.com/faq/cms&drl.htm, accessed on 22 May 2021). Furthermore, the raw data were common-average re-referenced. We combined EEG and GSR data from all of the sessions for the analysis.

### 2.2. EEG and GSR Processing

To remove a very slow electrical draft artifact, EEG data were zero-averaged and linearly detrended. Because EEG artifacts in the infraslow frequency band are not well-understood, no further EEG artifact reduction procedures were applied. Initially, the EEG and GSR signals were downsampled to a sampling rate of 8 Hz. This rate sufficiently detailed the ISA oscillatory phases while also accelerating calculations. We applied a surface Laplacian filter to the EEG signals to mitigate volume conduction effects, using the Current Source Density (CSD) toolbox [30,31,32]. The model parameters (m = 4 and λ = 10^−5^) were adopted from related EEG research [33,34]. To isolate ISA, the EEG and GSR signals underwent narrow-bandpass filtering centered at 10 different frequencies from 0.01 to 0.1 Hz, with an increment of 0.01 Hz. A finite impulse response (FIR) filter with Hamming window was used for narrow-bandpass filtering. The Hilbert transform was then performed to derive the oscillatory phases from ISA.

### 2.3. Phase Synchronization Index (PSI)

The arousal–ISA correlation was measured by phase synchronization between EEG ISA and GSR ISA. Let $\theta_{EEG}\left(t\right)$ and $\theta_{GSR}\left(t\right)$ be the oscillatory phases of EEG ISA and GSR ISA at time, t, respectively. An average phase difference between EEG and GSR over time (ΔΘ) was defined as follows:

(1) $$\Delta \Theta =\frac{1}{T}\sum_{t=1}^{T}\exp i\left(\theta_{EEG}\left(t\right)-\theta_{GSR}\left(t\right)\right),$$

where *T* denotes an experimental duration. ΔΘ can be rewritten as

(2) $$\Delta \Theta =A_{\Delta }\exp i\theta_{\Delta },$$

For some non-negative real numbers, $A_{\Delta }$, and oscillatory phase $\theta_{\Delta }$. A Phase Synchronization Index (PSI) was defined as the absolute value of ΔΘ, as follows:

(3) $$PSI=\left|\Delta \Theta \right|=A_{\Delta }.$$

**Figure 1:**
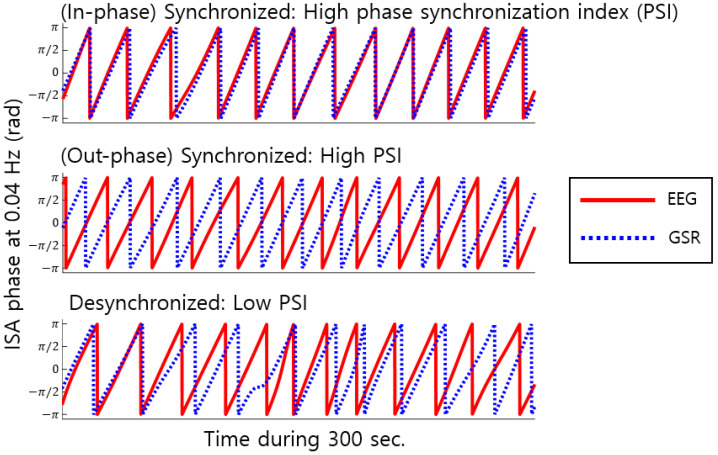
Phase synchronization measurement. Phase Synchronization Index (PSI) measures phase synchronization between the EEG (red solid) and GSR (blue dotted) signals. Illustrations of phase synchronization between EEG ISA and GSR ISA at an example frequency of 0.04 Hz are given. High values of PSI indicate that the phase differences between EEG and GSR are constant over time (**top** and **middle**). Low PSI values indicate variability in phase differences over time (**bottom**).

If the phase differences between EEG and GSR were constant over time, PSI had high values. Otherwise, PSI had low values (Figure 1). This definition of PSI is similar to the Phase-Locking Value (PLV) [35], but slightly different, such as the fact that PSI is an average over time [36], while PLV is an average over trials.

We also generate surrogate PSI. Let $\hat{\theta_{GSR}\left(t\right)}$ be the oscillatory phase of GSR ISA at randomized time, t′, which was obtained by randomly shuffling $\theta_{GSR}\left(t\right)$. Then, surrogate $\hat{\Delta \Theta }$ was given by

(4) $$\hat{\Delta \Theta }=\frac{1}{T}\sum_{t=1}^{T}\exp i\left(\theta_{EEG}\left(t\right)-\hat{\theta_{GSR}\left(t\right)}\right).$$

$\hat{\Delta \Theta }$ could be rewritten as

(5) $$\hat{\Delta \Theta }=A_{\hat{\Delta }}\exp i\theta_{\hat{\Delta }}.$$

Surrogate PSI was then defined as follows:

(6) $$\hat{PSI}=\left|\hat{\Delta \Theta }\right|=A_{\hat{\Delta }}.$$

$\hat{PSI}$ was used to construct a test statistic to evaluate the statistical significance of PSI. The mean Phase Difference (mPD) was defined as the oscillatory phase of ΔΘ, as follows:

(7) $$mPD=\theta_{\Delta },$$

where $\theta_{\Delta }$ is obtained from Equation (2).

### 2.4. Phase Difference Synchronization (mPD-PSI) Coupling

The mean Phase Difference (mPD) between EEG ISA and GSR ISA could vary from −π to π rad. As such, we examined whether EEG ISA and GSR ISA were more phase-synchronized for a specific mPD or not. To this end, we calculated PSI and mPD for each EEG channel per participant. By repeating this calculation for all channels and all participants in each group, we generated a population of mPD-PSI pairs. To evaluate whether PSI exhibited particularly high values at certain mPDs, that is, whether there was a presence of mPD-PSI coupling, we applied the method used for quantifying phase–amplitude coupling [37]. Specifically, we treated mPD as the phase and PSI as the amplitude. First, the range of mPD, −π to π (rad), was divided into 16 segments. We collected all mPD-PSI pairs corresponding to each segment and calculated the average PSI value within each segment. These 16 average PSI values were then normalized to have a sum equal to 1. The normalized average PSI values were treated as a probability distribution, $P_{PSI}$. The Kullback–Leibler divergence between $P_{PSI}$ and the uniform distribution $P_{U}$ was used for quantifying mPD-PSI coupling, which was given by

(8) $$mPD-PSI\;coupling=\sum_{m=1}^{16}P_{PSI}\left(m\right)\frac{P_{PSI}\left(m\right)}{P_{U}\left(m\right)},$$

where $P_{PSI}\left(m\right)$ is the m-th normalized average PSI and $P_{U}\left(m\right)=1/16$.

We also generated surrogate mPD-PSI coupling as follows. First, we randomly shuffled mPDs from all channels in all participants in each group to obtain surrogate mPD-PSI pairs. In the same way as above, let $\hat{P_{PSI}}$ be the probability distribution of the 16 normalized average PSI values from these surrogate mPD-PSI pairs. Then, surrogate mPD-PSI coupling was given by

(9) $$\hat{mPD-PSI\;coupling}=\sum_{m=1}^{16}\hat{P_{PSI}\left(m\right)}\frac{\hat{P_{PSI}\left(m\right)}}{P_{U}\left(m\right)}.$$

Surrogate mPD-PSI coupling was used to construct a test statistic to evaluate the statistical significance of mPD-PSI coupling.

### 2.5. Spatial Distribution of Phase Differences

If mPD-PSI coupling was present during meditation, it would mean that PSI was relatively higher in certain phase differences between GSR and ISA. This potential dependency of PSI on phase differences might inform the characteristics of interactions between EEG ISA and the arousal fluctuation during meditation. One way to investigate the characteristics is to explore the spatial distribution of phase differences, at which PSI was relatively high, over the brain. We computed the spatial distribution of phase differences akin to the previous study [12]. A difference between our study and the previous one is that the arousal fluctuation was measured by GSR in our study, whereas it was linked to respiratory variation in the previous study.

Let $\Delta \Theta_{k}$ be the average phase difference between EEG ISA and GSR ISA over time in a participant, k, at a certain channel, as defined in Equation (1). The mean $\Delta \Theta_{k}$ among participants was expressed as follows:

(10) $$\frac{1}{K}\sum_{k=1}^{K}\Delta \Theta_{k}=A_{\tilde{\Delta }}\exp i\theta_{\tilde{\Delta }},$$

where $A_{\tilde{\Delta }}$ is a non-negative real number and $\theta_{\tilde{\Delta }}$ is an oscillatory phase. The grand mean Phase Difference (gmPD) was defined as the oscillatory phase of $\frac{1}{K}\sum_{k=1}^{K}\Delta \Theta_{k}$, as follows:

(11) $$gmPD=\theta_{\tilde{\Delta }}.$$

The distribution of gmPDs over EEG channels represented the spatial distribution of phase differences.

### 2.6. Principal Gradient

After mapping the spatial distribution of gmPD on the brain, we interpreted it by comparing it to the principal gradient of the brain that has been known to represent functional organization of the brain with a reduced dimensionality [38]. In fact, the previous study has reported a close relationship between the spatial distribution of phase differences between the arousal fluctuation and ISA and the principal gradient [12]. To investigate this relationship in relation to meditation, we computed the principal gradient similarly to the previous study by Margulies et al. [38]. First, the functional connectivity between two channels was measured by the correlation of their band-passed EEG signals for each infraslow frequency band (0.01, 0.02, …, 0.1 Hz). A correlation of less than zero was set to zero. This non-negative functional connectivity was then used to compute the principal gradient, using diffusion embedding with an alpha value of 0.5. The script for computing diffusion embedding was sourced from the BrainSpace toolbox [39], available at http://github.com/MICA-MNI/BrainSpace (accessed on 2 June 2024). If two EEG channels had similar principal gradient values, it means that these channels were more strongly connected. Conversely, a large difference in the principal gradient values between two channels indicated a relatively weak connection.

### 2.7. Statistical Analysis

A permutation test was conducted to determine the significance of PSI (Equation (3)) for each channel in each participant. We generated 10,000 surrogate PSIs (Equation (6)) and determined whether PSI exceeded the 95% percentile of surrogate PSIs, setting the significance level at 0.05.

To assess a difference in the PSI between the Expert and Novice groups, we collected the PSI values from all EEG channels of all participants per group. We conducted a one-tailed Wilcoxon rank-sum test to ascertain whether the PSI in one group was significantly higher than that in the other group. The *p*-values were corrected by using a false discovery rate (FDR) over frequency bands. The reasons why we used the one-tailed test are as follows: (1) Our hypothesis expects that the EEG-GSR correlation was increased in the Expert group compared to the Novice group, and (2) the qualitative result showed that the EEG-GSR correlation was increased in the Expert group.

We also assessed a difference in the PSI between the groups for individual channels by collecting the PSI values of each group at each channel. Again, a one-tailed Wilcoxon rank-sum test was conducted to ascertain whether the PSI in a specific group was significantly higher, using various significance levels of 0.05 or lower.

A permutation test was performed to assess the significance of mPD-PSI coupling (Equation (8)) for each channel in each participant. We generated 10,000 surrogate mPD-PSI couplings (Equation (9)) and assessed them to ascertain whether mPD-PSI coupling exceeded the 95% percentile of surrogate mPD-PSI couplings with the significance level at 0.05.

The similarity between the spatial distribution of $\left|gmPD\right|$ and principal gradients was assessed using linear regression with the significance level at 0.05. Here, we used the absolute value of gmPD, which is non-circular, rather than gmPD, because the principal gradient was non-circular.

## 3. Results

**Figure 2:**
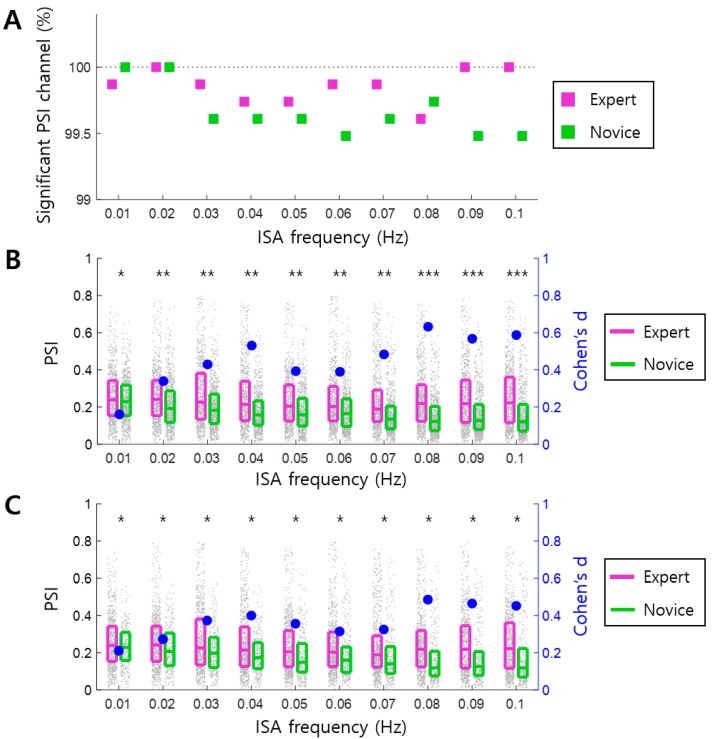
Arousal–ISA correlation in Expert and Novice groups. (**A**) The ratio of EEG channels showing significant PSI values (permutation test, *p* < 0.05) is presented for 10 different infraslow frequency bands (0.01~0.1 Hz incremented by 0.01 Hz), for each of the Expert and Novice groups. All of the ratios are above 99%. (**B**) Differences in the PSI between Expert and Novice groups are illustrated for each infraslow frequency band. There are N = 768 PSI values in the Expert and Novice groups, respectively. (**C**) Similar to (**B**), but the Novice group includes male or female, with age under 38 years old. There are N = 768 and 320 PSI values in the Expert and Novice groups, respectively. In (**B**,**C**), each gray dot represents the PSI value of each EEG channel in each participant. The three horizontal lines in a box represent the 25th, 50th, and 75th percentiles, respectively. Asterisks indicate a significant difference in the PSI between the Expert and Novice groups (one-tailed Wilcoxon rank-sum tests, *, *p* < 0.05; **, *p* < 10^−10^; ***, *p* < 10^−20^, all *p*-values are FDR-corrected). Each blue circle represents Cohen’s d, which indicates the effect size.

First, we verified the presence of arousal–ISA correlations by evaluating the significance of PSI values between EEG ISA and GSR ISA. We observed that the PSI was significantly higher than chance for all infraslow frequencies (permutation test, *p* < 0.05), at over 99% of EEG channels of all participants in both groups, demonstrating clear arousal–ISA correlations (Figure 2A).

Next, we assessed whether arousal–ISA correlations differed between the Expert and Novice groups. We observed that PSI was significantly higher in the Expert group compared to the Novice group for all frequency bands (one-tailed Wilcoxon rank-sum test, N = 768, FDR-corrected *p* < 10^−10^ for all frequency bands except for 0.01 Hz, where FDR-corrected *p* < 0.05). The effect size (Cohen’s d) exceeded 0.2 for all frequencies except 0.01 Hz. In 0.04, 0.08, 0.09, and 0.1 Hz, the effect size exceeded 0.5 (Figure 2B). In female participants with the age of menopause or perimenopause, increased skin sensitivity can make the GSR measurement unstable, potentially leading to autonomic nervous misbalances. Furthermore, the Novice group here included many female participants compared to the Expert group. In order to improve the gender balance between the two groups, and to exclude the issue of autonomic nervous misbalances in elder female participants, we excluded female participants with an age of over 38 years old. The revised results still showed that PSI was significantly higher in the Expert group compared to the Novice group for all frequency bands (one-tailed Wilcoxon rank-sum test, N = 768 and 320, FDR-corrected *p* < 0.05). Effect sizes (Cohen’s d) exceeded 0.2 for all frequencies (Figure 2C).

**Figure 3:**
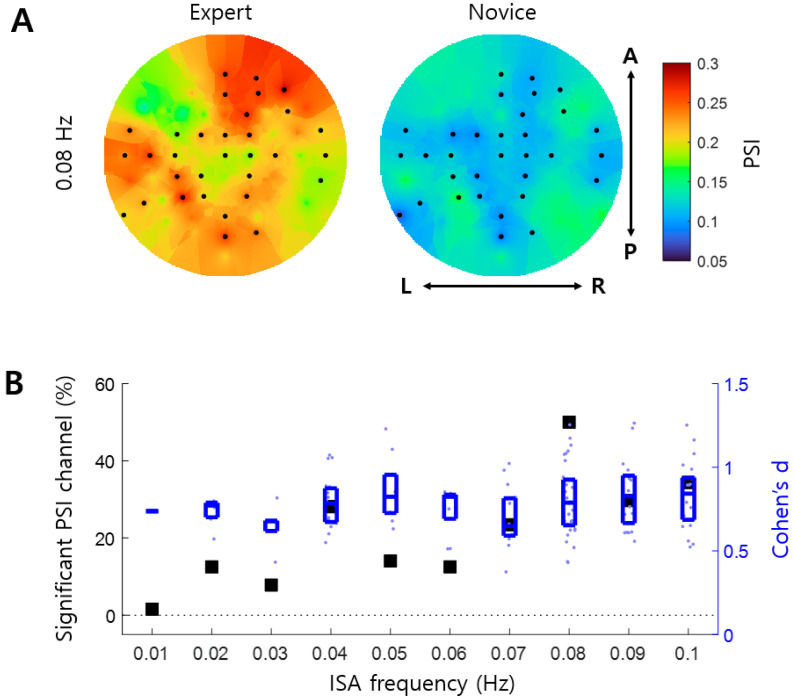
Spatial distribution of arousal–ISA correlation in the Expert and Novice groups. (**A**) Spatial distribution of PSI at 0.08 Hz. Each dot indicates the EEG channels that exhibit higher Phase Synchronization Index (PSI) values in the Expert group than the Novice group (one-tailed Wilcoxon rank-sum test, *p* < 0.05). A, P, L, and R indicate anterior, posterior, left, and right, respectively. (**B**) Ratios of the EEG channels at which PSI is significantly higher in the Expert group than in the Novice group (one-tailed Wilcoxon rank-sum test, *p* < 0.05). Each light blue dot represents the Cohen’s d-value of each significant EEG channel. The three horizontal lines in a box represent the 25th, 50th, and 75th percentiles, respectively.

Furthermore, we examined the group difference of PSI for individual EEG channels in order to investigate whether the group difference was localized in a certain brain region. We first visually verified that there was no specific pattern of the spatial distribution of PSIs (Figure 3A). At 21.41 ± 14.51% of EEG channels on average across frequency bands, the PSI values of the Expert group were significantly higher than those of the Novice group. We found no EEG channel at which the PSI values of the Expert group were significantly lower than those of the Novice group (one-tailed Wilcoxon rank-sum test, N = 12, *p* < 0.05). The effect size (Cohen’s d) for the significant EEG channels was 0.79 ± 0.19 on average (Figure 3B).

**Figure 4:**
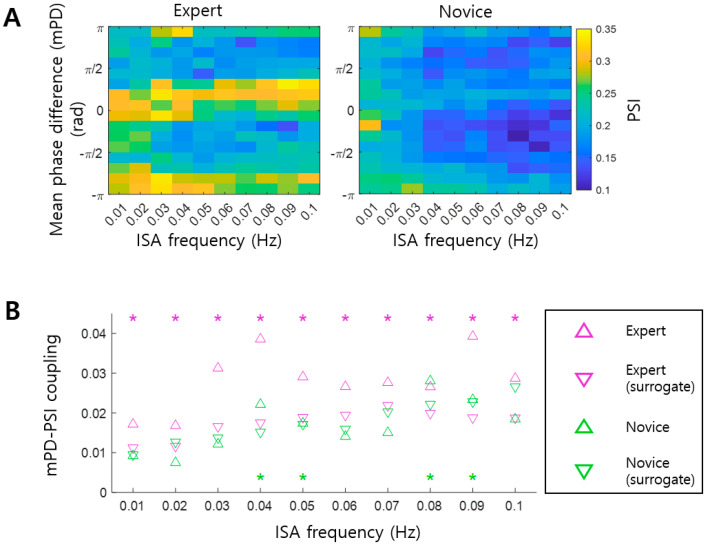
Coupling of phase synchronization with phase differences between EEG ISA and GSR. (**A**) PSI is illustrated as a function of mean Phase Difference (mPD) and ISA frequency. In the Expert group, the PSI tends to be high when the mPD between EEG and GSR is near 0 or π rad (**left**). In the Novice group, however, no such tend appears clearly (**right**). (**B**) Coupling between mPD and PSI is shown. Upward triangles represent the estimate of mPD-PSI coupling (see Equation (8) in the text). Downward triangles represent the 99.9% confidence level of mPD-PSI coupling using a surrogate method (see Equation (9) in the text). Asterisks with different colors indicate significant mPD-PSI coupling for each group (permutation test, *p* < 0.001).

We investigated whether enhanced arousal–ISA correlations in the Expert group were associated with specific phase differences between EEG ISA and GSR ISA. PSI tended to be high when the mean Phase Difference (mPD) between EEG and GSR was near 0 or π radians in the Expert group (Figure 4A). In contrast, we observed no clear tendency in the Novice group (Figure 4A). This indicates that phase synchronization between EEG and GSR became stronger when the phases were either in-phase or antiphase. Such mPD-PSI coupling was significant at all infraslow frequencies in the Expert group (permutation test, *p* < 0.001) (Figure 4B). In comparison, mPD-PSI coupling in the Novice group was significant only at four frequencies, including 0.04, 0.05, 0.08, and 0.09 Hz (Figure 4B).

**Figure 5:**
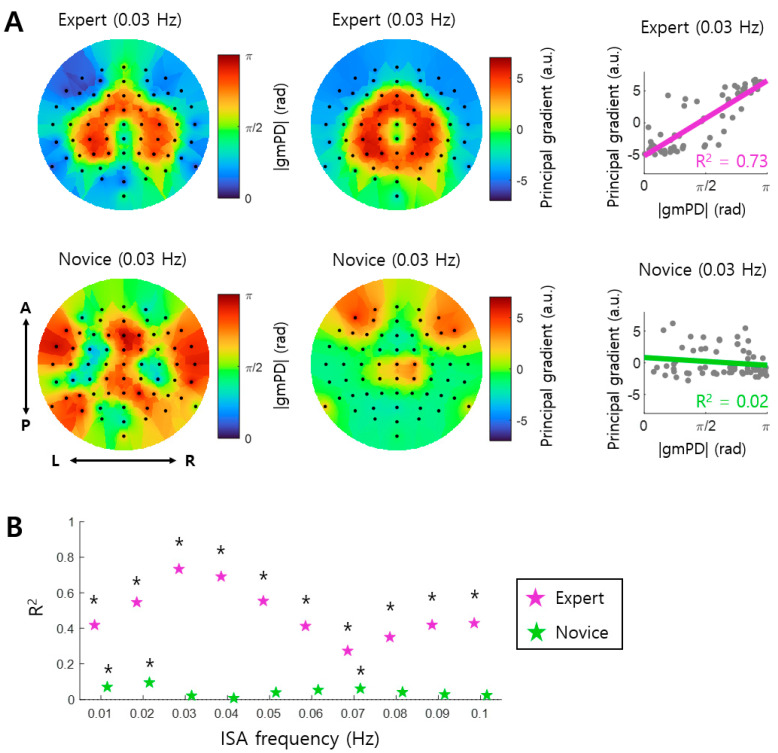
Spatial distribution of phase differences represents functional organization. (**A**) The topographies of the absolute values of the grand mean Phase Difference ($\left|gmPD\right|$) (**left**) and the principal gradient (**right**) within an example infraslow frequency band (0.03~0.04 Hz) are shown for the Expert and Novice groups, respectively. In these topographies, each dot represents an EEG channel. A linear relationship between $\left|gmPD\right|$ and the principal gradient across channels is assessed by linear regression analysis (**right**). Here, each gray dot represents an EEG channel, and the bold line indicates the linear fit across EEG channels. A: anterior; P: posterior; L: left; and R: right. (**B**) The degree of linear correlation between $\left|gmPD\right|$ and the principal gradient for each infraslow frequency band is represented by the coefficient of determination (R^2^). Black asterisks mark significant linear correlations (linear regression, *p* < 0.05).

After finding significant mPD-PSI coupling, especially in the Expert group, we further explored the characteristics of interactions between EEG ISA and GSR by mapping the spatial distribution of phase differences on the brain. Specifically, we constructed the topography of the absolute value of grand mean Phase Differences, $\left|gmPD\right|$, over EEG channels for each infraslow frequency band (see left of Figure 5A for an example at 0.03 Hz). We confirmed that most $\left|gmPD\right|$ values were near 0 and π rad, as shown in Figure 4A. We also observed that EEG channels exhibiting $\left|gmPD\right|$ values of 0 and π rad formed spatial clusters (Figure 5A, left). Similarly, we constructed the topography of the principal gradients over EEG channels for each infraslow frequency band (see middle of Figure 5A for an example at 0.03 Hz). Notably, the topography of the principal gradients resembled that of $\left|gmPD\right|$. This observation was quantified by a linear regression analysis, from which we found that the spatial distribution of $\left|gmPD\right|$, and that of the principal gradient, were linearly correlated in the Expert group at all infraslow frequency bands (Figure 5B) (*p* < 0.05; see right of Figure 5A for an example at 0.03 Hz). In contrast, linear correlations in the Novice group were much lower than those in the Expert group, with significant correlations observed only at three frequency bands, including 0.01, 0.02, and 0.07 Hz (Figure 5B; see Figure 5A for an example at 0.03 Hz). Thus, the phase differences of 0 and π radians appeared to be related to functional organization, represented by the spatial distribution of principal gradients. Since the brain signals at the channels that show similar principal gradients are similar to each other (Margulies et al., 2016 [38]), high correlations between the principal gradient and $\left|gmPD\right|$ in the Expert group indicated that EEG ISAs with similar phase differences to GSR ISA (near 0 or π rad) were similar to each other. Additionally, since the spatial distribution of the principal gradient represents functional organization of the brain (Margulies et al., 2016 [38]), EEG ISAs in the Expert group were spatially organized according to functional organization, along with systematic phase differences from GSR ISA.

## 4. Discussion

Correlations between arousal and infraslow activity (ISA) reflect the global organization of CNS and ANS. However, it remains unclear whether this arousal–ISA correlation is altered by meditation. This study inspected EEG and GSR data recorded during meditation. Changes in arousal–ISA correlation were assessed through EEG-GSR phase synchronization. We observed arousal–ISA correlations in both groups, with experienced meditators exhibiting higher correlations than novices (Figure 2 and Figure 3). In experienced meditators, increased arousal–ISA correlation was evident at specific oscillatory phase differences (0 and π radians) between EEG ISA and GSR (Figure 4). Moreover, in experienced meditators, the spatial distribution of phase differences was correlated with that of the principal gradient (Figure 5). Hence, meditation increases correlations between the arousal fluctuation and ISA by making their phases more synchronized, through which the spatial organization of ISA is more likely to reflect functional organization of the brain.

Increased arousal–ISA correlations in experienced meditators (Figure 2 and Figure 3) suggest enhanced global organization of the nervous systems. This refers specifically to the integration of CNS (EEG) with ANS (GSR). Such global organization may underpin the positive effects of meditation. For instance, meditation is known to alleviate mental disorders, including major depressive disorder [5,6], which is associated with increased global organization of brain signals across various brain regions [19,20,21]. Likewise, improved cognitive function is another benefit of meditation [1], where global organization of brain signals from multiple regions enhances cognitive capabilities [40,41]. Therefore, global organization can be a pivotal mechanism behind the beneficial effects of meditation. What distinguishes this study from previous research is its focus on global organization between CNS and ANS via EEG ISA and GSR. The observed strengthening of arousal–ISA correlations at specific oscillatory phase differences between EEG and GSR (Figure 4) indicates that this global organization occurs through specific phase differences (0 and radians) between the ISAs of CNS and ANS.

In experienced meditators, we observed that the brain regions were organized according to phase differences between EEG ISA and GSR ISA. In other words, the brain regions with small phase differences near 0 rad, and those with large phase differences near π rad, form distinct spatial clusters (Figure 5A). Remarkably, these clusters largely corresponded to the clusters of the brain regions formed by the principal gradient that generally represents functional organization of the brain [38]. This finding suggests that meditation enhances synchronization between the arousal fluctuation and ISA, which may reshape global functional organization of the brain. We surmise that this brain organization by meditation may occur through phase-locking between the arousal fluctuation and ISA, such that brain regions with similar functional properties (i.e., similar principal gradients) share phase-locking of ISA to the arousal fluctuation with a particular phase difference (e.g., 0 or π rad). Since ISA reflects neural excitability, a possible neural mechanism underlying meditation would be to orchestrate global neural excitability in the brain in a more organized manner through synchronizing ISAs of various brain networks with the arousal fluctuation at difference phases.

**Figure 6:**
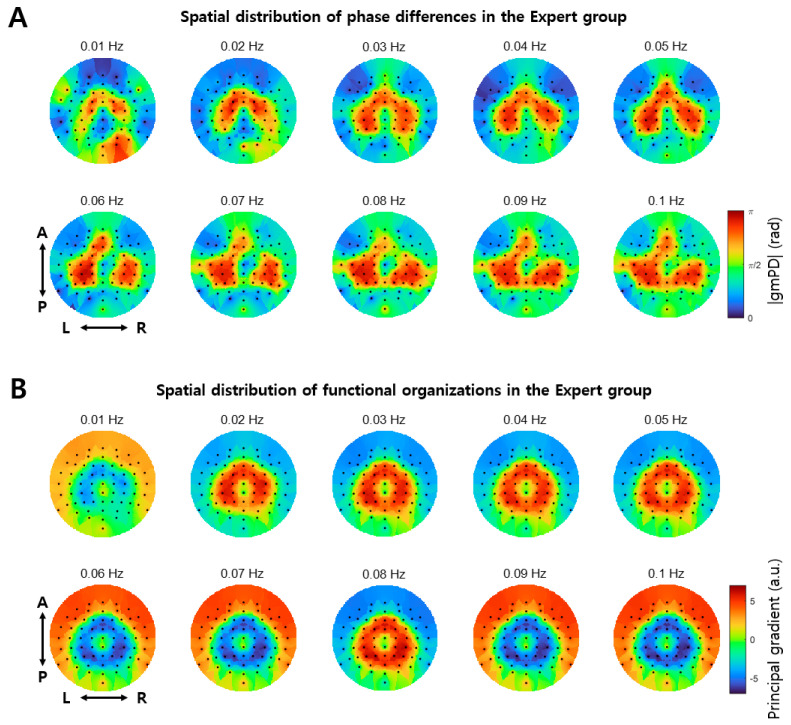
Spatial distribution of phase differences and functional organizations in the Expert group. (**A**) The topographies of the absolute values of the grand mean Phase Difference ($\left|gmPD\right|$) in various infraslow frequency bands for the Expert group. (**B**) The topographies of the principal gradient in various infraslow frequency bands for the Expert group. In both (**A**) and (**B**), A: anterior; P: posterior; L: left; and R: right. In these topographies, each dot represents an EEG channel.

The functional organization of the brain for an infraslow frequency of 0.03 Hz in Figure 5 displayed specific spatial clusters, with a donut-shaped cluster at the central region, and another cluster at outer regions, only in the Expert group. This specific distribution pattern was common over different infraslow frequencies in the Expert group (Figure 6). In fact, the spatial distributions of the principal gradient and the grand mean Phase Difference were very similar across different infraslow frequencies, which indicates that the functional organizations of the brain over all infraslow frequencies share a common pattern.

In the Expert group, phase differences between EEG ISA and GSR were the largest in pericentral brain regions over different infraslow frequencies (Figure 6A). Moreover, these regions exhibited similar EEG ISAs, revealing the specific functional organization (Figure 6B). In fact, in experienced meditators, the amplitude of fast oscillations, such as beta or gamma oscillations, were reduced in these regions [42,43]. Since the phase of ISA and the amplitude of fast oscillations are coupled [10,11], we conjecture that the largest phase difference between EEG ISA and GSR may be related to the reduced amplitude of fast oscillations in these regions. As prior research suggests that the reduced amplitude in these regions can indicate the attentive brain state or the embodied sense of self [42,43], the largest phase difference between EEG ISA and GSR in these regions may also reflect the same brain state and function. From these interpretations, we speculate that the attentive brain state during meditation may be supported by a neurophysiological substrate that neural excitabilities of the central (EEG ISA) and autonomic (GSR) nervous systems alternate with certain phase differences, such that net excitability sustains this state in experienced meditators.

In this study, we assessed correlations between the arousal fluctuation and ISA via phase synchronization of the ISA of GSR and the ISA of EEG. In other words, we examined phase-to-phase coupling between the ISAs of GSR and EEG. It might also be plausible to inspect phase-to-amplitude coupling by examining the distribution of the GSR amplitude over different phases of EEG ISA. However, we did not examine such phase-to-amplitude coupling in this study because we mainly focused on the relationship between temporal fluctuations of ISA and arousal, which can be more directly revealed by phase-to-phase coupling.

EEG ISA can be viewed as a reflection of neuronal excitability fluctuations, as it is correlated with the amplitude of fast EEG oscillations, which are indicative of neuronal excitability [10,11,44,45,46]. Our previous study showed that experienced meditators exhibit reduced EEG ISA amplitude and a decreased correlation with the amplitude of fast EEG, indicative of reduced neuronal excitability [18]. This suggests that experienced meditators are less sensitive to intrinsic neuronal excitability fluctuations. Given that arousal is also a type of excitability, this could be interpreted as diminishing the influence of arousal fluctuations. However, the reduced influence of arousal fluctuations does not necessarily weaken arousal–ISA correlation. Therefore, our findings in the present study are compatible with the global organization hypothesis, as evidenced by an increase in arousal–ISA correlations.

### 4.1. Limitations

A limitation of this study is that it only includes results for specific types of meditation. We used a dataset on Himalayan yoga meditation [28], which is classified as a focused attention meditation practice [47]. However, meditation practices can also involve open monitoring, a combination of both types [48,49], or other types of the meditation category, e.g., the meditation category classified based on EEG responses during meditation [50]. Therefore, future research may explore arousal–ISA correlation across various types of meditation to provide more comprehensive understanding.

The limited sample size of this study highlights the need for follow-up studies with larger sample sizes to generalize the findings. Additionally, the gender and age were not completely balanced across the groups, which limited the rigorous comparisons between groups. Future studies should aim to address these issues to enable more rigorous conclusions.

### 4.2. Conclusions

To investigate changes in arousal–ISA correlation, this study analyzed a publicly available dataset containing EEG data recorded from both experienced meditators and novices during meditation sessions. Arousal fluctuations were measured through GSR, and changes in arousal–ISA correlation were explored through EEG-GSR phase synchronization. Arousal–ISA correlations were observed in both groups, with experienced meditators exhibiting higher correlations than novices. Specifically, in experienced meditators, an increased arousal–ISA correlation was evident at specific oscillatory phase differences between EEG and GSR. Furthermore, brain regions in experienced meditators showing similar EEG-GSR phase differences corresponded to brain regions clustered based on functional organization of the brain. This study indicates that the increased arousal–ISA correlations in experienced meditators are associated with the enhanced global organization of the nervous system, suggesting that the positive effects of meditation might be mediated through such organization.

## Data Availability

The analysis codes supporting the conclusions of this article will be made available by the authors upon request, due to legal reasons.
